# Correction to “Functional Relationships of Two NFU Proteins in Maintaining the Abundances of Mitochondrial Iron–Sulfur Proteins”

**DOI:** 10.1002/pld3.70116

**Published:** 2025-10-28

Zhao, J. , Satyanarayan, MB , VanSlambrouck JT , Kolstoe AJ , Voyt MJ , Jamesa GO , Yu F , & Lu Y (2025). Functional relationships of two NFU proteins in maintaining the abundances of mitochondrial iron–sulfur proteins. Plant Direct, 9: e70081. 10.1002/pld3.70081.40443787 PMC12120262

The last name of the sixth author was incorrect. It should be “James,” not “Jamesa.”

This spelling has been corrected in the published article on Wiley Online Library.

We apologize for this error.

